# Attrition during pre-ART and ART time periods among adolescents enrolled in Integrated HIV Care Programme in Myanmar, 2005–2017

**DOI:** 10.1017/S0950268819000906

**Published:** 2019-05-29

**Authors:** T. Htun, K. W. Y. Kyaw, T. K. Aung, J. Moe, A. A. Mon, C. L. Tun, O. Mon, S. Satyanarayana, H. N. Oo

**Affiliations:** 1 International Union against Tuberculosis and Lung Disease (The Union), Mandalay, Myanmar; 2 Centre for Operational Research, International Union against Tuberculosis and Lung disease (The Union), Paris, France; 3 Department of Paediatrics, University of Medicine, Mandalay, Myanmar; 4 Department of Public Health, National AIDS Programme, Ministry of Health and Sports, Myanmar

**Keywords:** ALHIV, death, loss to follow-up, LTFU, retention

## Abstract

Retaining adolescents (aged 10–19 years), living with HIV (ALHIV) on antiretroviral therapy (ART) is challenging. In Myanmar, 1269 ALHIV were under an Integrated HIV Care (IHC) Programme by June 2017 and their attrition (death and lost to follow-up) rates were not assessed before. We undertook a cohort study using routinely collected data of ALHIV enrolled into HIV care from July 2005 to June 2017 and assessed their attrition rates in June 2018 by time-to-event analysis. Of 1269 enrolled, 197(16%) and of 1054 initiated ART, 224 (21%) had an attrition defining event. The pre-ART and ART attrition rates were 21.8 (95% CI 19.0–25.1) and 6.4 (95% CI 5.6–7.3) per 100 person-years follow-up, respectively. The factors ‘at enrolment’ that were associated with higher hazards of attrition were: (1) WHO stage 3 or 4; (2) haemoglobin <10 gm/dl; (3) no documented CD4 cell counts, hepatitis B and C test results; and (4) injection drug use. Baseline hazards were high during the initial 1–2 years and after 5–6 years. The pre-ART and ART attrition rates in ALHIV were lower than those in Africa but higher than the children under IHC. This warrants designing and implementing additional care tailored to the needs of ALHIV under IHC.

## Introduction

Globally, in 2017, 36.9 million people (95% CI 31.1–43.9 million) were living with HIV [1]. Of these, it is estimated that about 1.8 million (95% CI 1.1–2.6 million) are adolescents (aged 10–19 years) living with HIV [2]. Compared with older adults, adolescents and young adults (aged 15–24 years) have a higher loss to follow-up (LTFU) rates in seven African countries [3]. When compared to paediatric HIV, adolescents living with HIV (ALHIV) were at higher risk of delay in treatment initiation, late presentation and LTFU [4].

The retention of ALHIV in HIV care programme is challenging and is dependent on many factors including barriers to access health care, lack of psychological support, lack of trained health care workers on adolescent health care issues and lack of support during transition from paediatric to adult care [4, 5]. A study in South Africa reported that community-based support to ALHIV can reduce mortality and LTFU [6]. Mortality in adolescents living with peri-natally acquired HIV remains substantially higher [7]. In a study in Thailand, after the initial 6 months of antiretroviral therapy (ART), being an adolescent, persistent viraemia, poor nutritional status and severe anaemia were associated with poor clinical outcomes [8].

In Myanmar, the prevalence of HIV in adults aged 15–49 is 0.7% (95% CI 0.6–0.9) by 2017 [2]. There were about 220 000 (95% CI 200 000–260 000) people living with HIV (PLHIV) in 2017 and the ART coverage among all PLHIV is 66% and children aged 0–14 years is 91% [1]. In 2017, the estimated number of ALHIV in Myanmar was 10 000 (95% CI 7900–14 000) [2]. Since 2005, The Union (an International Non-Government Organization) has been implementing an Integrated HIV Care (IHC) Programme in close collaboration with National AIDS Programme (NAP) in 37 townships through 16 ART centres and 33 decentralised ART sites. The HIV care consists of paediatric care (for those aged <15 years) and adult care (for those aged ⩾15 years). By December 2017, there were more than 29 000 PLHIV receiving ART care under the IHC Programme (~20% national ART cohort). Among them about 1000 were adolescents at the ‘time of enrolment’ into care since inception of the IHC Programme.

Although the IHC Programme routinely monitors the outcomes of those on paediatric and adult care, there is lack of separate focus in monitoring the outcomes of adolescents despite the fact that they are at higher risk of attrition (LTFU, death). A previous study done in Myanmar reported that adolescents have 2–3 times higher risk of attrition and/or virological failure when compared to adults living with HIV [9]. However, within the adolescent age group, what factors were associated with attrition are unknown. There are no specific guidelines for HIV counselling, treatment and care for adolescents in Myanmar.

In this study, we assessed the magnitude and factors associated with attrition prior to ART initiation (also called pre-ART attrition) and attrition after ART initiation (also called ART attrition) among adolescents enrolled for HIV care under the IHC Programme in Myanmar between 2005 and 2017.

## Methods

### Study design

This is a cohort study using routinely collected data under the IHC Programme in Myanmar.

### Setting

#### General setting

Myanmar is one of the South-East Asian countries bordered in the north and northeast by China, the east and southeast by Laos and Thailand, the south by the Andaman Sea and the Bay of Bengal and on the west by Bangladesh and India. The population of Myanmar was 54 million in 2018 [10]. According to the Myanmar Census data in 2014, 30% of the population living in urban areas and 9.7 million are adolescents aged 10–19 years [11].

#### Specific setting

People living with HIV who are tested and diagnosed as HIV in NAP, outpatient clinic and inpatient ward of public hospitals and tuberculosis outpatient departments of different townships in five States and Regions (Mandalay, Sagaing, Magway, Yangon and Shan State) are enrolled into the IHC Programme.

A unique identifier is provided to each enrolled patient. HIV care is broadly divided into Paediatric care for those aged <15 years (delivered through paediatricians at the public hospitals) and adult care for those aged ⩾15 years (delivered through physicians of the public hospitals assisted by medical officers employed by The Union). Enrolment of ALHIV into paediatric or adult care is not strict based on age especially for those aged 13–16 years. For them, depending on the knowledge, awareness and disclosure status, ALHIV are enrolled (prior to 15 years or later than 15 years) into the adult cohorts. The care, ART initiation and treatment monitoring are in accordance with the WHO and National guidelines which have changed in 2006 [12, 13], 2011 [14, 15], 2014 [16] and 2017 [17]. When the child reaches the adolescent age (~10–12 years), paediatrician and medical social workers start partial disclosure counselling as well as provide psychosocial support as an initial step for preparing transition to adult care. In a few centres, adolescents also participate in youth club activities, which additionally give them information on HIV disease and life skills training. The patient flow and treatment monitoring processes under the IHC Programme have been described in previous studies of The Union [9, 18]. At every visit, the medical officers fill out all the information into the standardised visit forms and trained data assistants transcribe the paper-based data to the electronic database created using PHP (Hypertext Preprocessor) and MySQL (My Structured Query Language).

### Study population and sites

Adolescents (i.e. PLHIV aged 10–19 years) at the time of enrolment into IHC Programme from July 2005 to June 2017 at 16 ART centres and 33 decentralised ART sites in 37 townships where IHC Programme is being implemented in Myanmar.

### Data variables, sources of data and data collection

We collected the following information from the electronic database maintained at the IHC: date of birth/age at enrolment, IHC code, outcome (regular follow-up, death, LFTU, transfer out), outcome date, enrolment date, ART initiation date, age, sex, route of HIV transmission, ART site, baseline WHO staging, baseline CD4 count, initiated ART regimen, hepatitis B, hepatitis C infection at baseline, baseline haemoglobin gm/dl.

### Analysis and statistics

The data from electronic databases were imported into STATA version 14.2 (College Station, TX, USA).

We have used numbers and proportions for summarizing categorical variables. We have calculated the attrition rate per 100 person-years of follow-up for pre-ART and ART care periods.

We have calculated the follow-up period for pre-ART from enrolment date to outcome date for the outcome death and LTFU if ART was not started or up to ART start date if ART was started. The censor date (30 June 2018) was used as end of follow-up date for adolescents who were regular on follow-up and not started on ART.

The follow-up period for ART period was calculated from ART initiation date to outcome date (death and LFTU). The censor date of 30 June 2018 was used for adolescents who are on regular follow-up.

We have used the Cox-proportional hazard model to study the association between various demographic and clinical factors with attrition during pre-ART and ART periods. Proportionality assumptions were tested using Shenfield residuals, log–log plots and observed *vs.* predicted survival plots. Factors with a *P* value <0.05 was considered as statistically associated with attrition.

### Ethics approval

We obtained ethics approval from the Ethics Advisory Group of The Union, Paris, France and the ethical review committee of the Department of Medical Research, Ministry of Health and Sports, Myanmar. We also obtained permission and support for the Operational Research (OR) from the NAP. As this study involved the review of existing programme records, we received a waiver from obtaining informed consent from patients from both ethical committees.

## Results

**Fig. 1.**
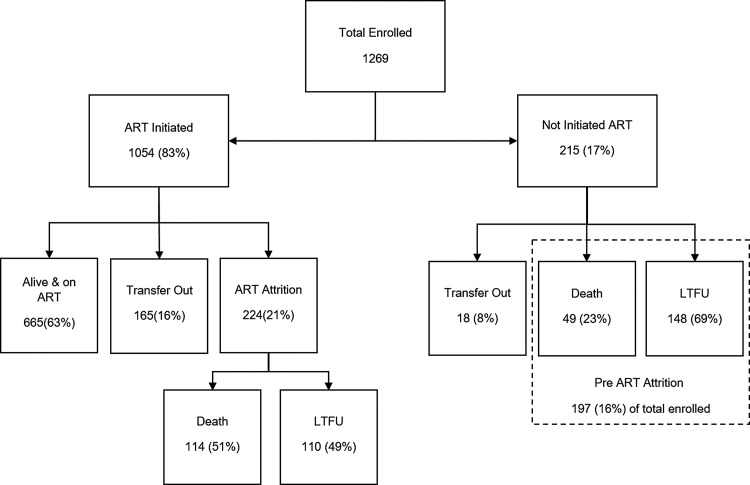
Flow diagram showing the ART initiation, attrition and disaggregation of attrition among the adolescents enrolled in IHC Programme from July 2005 to June 2017. ART, antiretroviral therapy; LTFU, loss to follow-up; IHC, Integrated HIV Care Programme.

From March 2005 to June 2017, 1269 adolescents were enrolled in the ART programme. Of them, 1054 (83%) were initiated on ART by the censor date (30 June 2018). Of the 215 (23%) patients who were not initiated ART, 148 (69%) were LTFU, 49 (23%) had died and 18 (8%) were transferred-out to other ART care facilities. Of the 1054 who were initiated on ART, 665 (63%) were alive and on ART as on the censor date and 165 (16%) were transferred to other care facilities. The remaining 224 (21%) were either dead or lost to follow-up (ART attrition) (Fig. 1). The crude 1-, 2- and 5-year retention rate of those initiated on ART was 88.7%, 84.8%, and 74.2% respectively (Table 1).

**Table 1.** Yearly retention of adolescents who were initiated ART in IHC Programme, Myanmar from July 2005 to June 2017

Year	Total (*n*)	Retention (*n*)	Retention (%)	95% CI
1	1054	938	88.7	(87–90)
2	877	842	84.8	(82–87)
3	681	656	81.3	(79–84)
4	501	487	78.7	(76–81)
5	370	352	74.2	(71–77)

CI, confidence interval; IHC, Integrated HIV Care Programme; ART, antiretroviral therapy.

### Pre-ART attrition rates and factors associated with pre-ART attrition

**Fig. 2.**
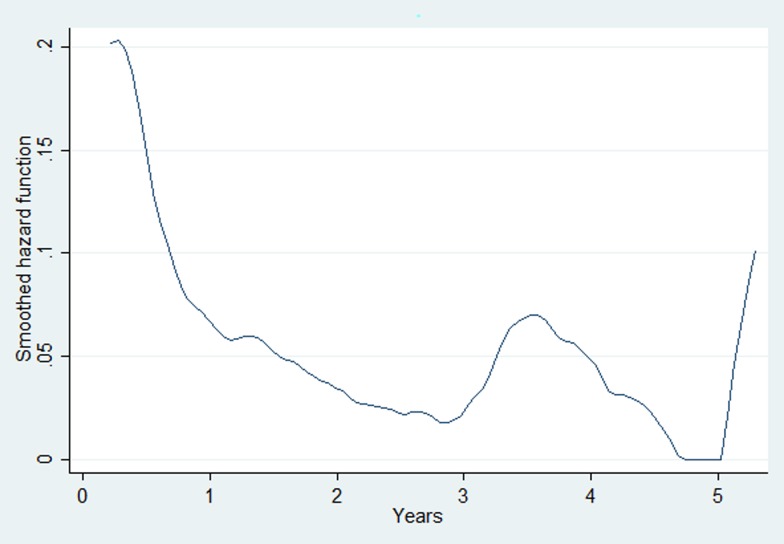
The baseline hazard function for pre-ART attrition derived from the multivariable Cox-proportional hazards model for the adolescent cohort enrolled in the Integrated HIV Care Programme, Myanmar (2005–2017). ART, antiretroviral therapy; HIV, human immunodeficiency virus.

The overall attrition rate during the pre-ART period was 21.8 (95% CI 19.0–25.1) per 100 person-years of follow-up. ALHIV who revealed their risk for HIV transmission as intravenous drug user and blood transfusion, those who had baseline haemoglobin <10 gm/dl and those without documented CD4 cell count test, hepatitis B and C test and haemoglobin test results at enrolment had higher hazards of attrition during the pre-ART period (Table 2). The baseline hazard function for pre-ART attrition derived from the multivariable Cox-proportional hazards model is given in Figure 2. This indicates that the hazard for pre-ART attrition was highest during the initial 6–12 months of enrolment followed by secondary rise after 3 and 5 years.

**Table 2.** Socio-demographic and clinical characteristics associated with pre-ART attrition among adolescents enrolled in IHC Programme, Myanmar from July 2005 to June 2017

Description	Total *n* (%)	Attrition *n* (%)	Attrition rate (100 py) (95% CI)	Unadjusted hazard ratio (95% CI)	Adjusted hazard ratio (95% CI)
Total	1269 (100)	197 (16)	21.8 (19.0–25.1)		
Sex					
Male	656 (52)	110 (17)	26 (21.6–31.4)	1.2 (0.9–1.6)	
Female	613 (48)	87 (14)	18.1 (14.7–22.4)	reference	
Entry cohort					
Adult	669 (53)	139 (21)	28.6 (24.2–33.8)	2.1 (1.6–2.9)**	1.3 (0.6–2.6)
Paediatric	600 (47)	58 (10)	13.9 (10.8–18)	reference	reference
Age at enrolment					
10–14 years	650 (51)	69 (11)	14.4 (11.4–18.3)	reference	reference
15–19 years	619 (49)	128 (21)	30.2 (25.4–35.9)	2 (1.5–2.7)**	0.8 (0.4–1.5)
Risk factor					
Heterosexual	261 (21)	57 (22)	25 (19.3–32.4)	2.1 (1.5–3)**	1.3 (0.6–2.9)
MSM	17 (1)	4 (24)	75 (28.2–199.9)	3.2 (1.2–8.9)*	4.3 (1.5–12.3)
FSW	7 (1)	3 (43)	45.2 (14.6–140.1)	3.2 (1–10.2)*	1.8 (0.7–4.9)
IDU	77 (6)	27 (35)	40.8 (28–59.5)	3.2 (2.1–5)**	3.3 (1.3–8.6)*
Blood Transfusion	50 (4)	11 (22)	33.1 (18.3–59.8)	2.5 (1.3–4.8)*	2.4 (1.2–4.9)*
MTCT	715 (56)	68 (10)	13.6 (10.8–17.3)	reference	reference
Unknown	142 (11)	27 (19)	42.2 (28.9–61.5)	2.4 (1.6–3.8)**	1.8 (1.0–3.4)
Baseline WHO stage					
Stage 1	307 (24)	51 (17)	21.3 (16.2–28)	reference	reference
Stage 2	271 (21)	38 (14)	15.9 (11.6–21.9)	0.8 (0.5–1.2)	1.0 (0.7–1.6)
Stage 3	535 (42)	72 (13)	20.1 (15.9–25.3)	0.9 (0.6–1.3)	1.0 (0.7–1.6)
Stage 4	146 (12)	30 (21)	47.2 (33–67.5)	1.7 (1.1–2.7)*	1.6 (1.0–2.4)
Not recorded	10 (1)	6 (60)	375.1 (168.5–834.9)	8.4 (3.6–19.6)**	2.1 (0.8–5.5)
Baseline CD4 (/mm3)					
<200	494 (39)	38 (8)	14.5 (10.5–19.9)	1.1 (0.7–1.7)	1 (0.6–1.7)
200–349	269 (21)	13 (5)	8.1 (4.7–13.9)	0.6 (0.3–1.1)	0.6 (0.3–1.2)
350–499	160 (13)	19 (12)	12.1 (7.7–18.9)	1 (0.6–1.7)	1 (0.5–1.8)
⩾500	226 (18)	32 (14)	11.2 (7.9–15.8)	reference	reference
Not recorded	120 (9)	95 (79)	271.2 (221.8–331.6)	14.6 (9.7–22.1)**	3.2 (1.3–7.6)*
Hepatitis B antigen					
Positive	66 (5)	7 (11)	12.8 (6.1–26.9)	1.1 (0.5–2.4)	1.2 (0.8–1.9)
Negative	974 (77)	90 (9)	12 (9.8–14.8)	reference	reference
Not recorded	229 (18)	100 (44)	99.8 (82–121.4)	6.1 (4.6–8.1)**	2.1 (1.2–3.5)*
Hepatitis C antibody					
Positive	59 (5)	11 (19)	16.1 (8.9–29.1)	1.6 (0.8–3)	0.9 (0.4–2.0)
Negative	981 (77)	86 (9)	11.7 (9.5–14.5)	reference	reference
Not recorded	229 (18)	100 (44)	99.8 (82–121.4)	6.3 (4.7–8.5)**	2.1 (1.2–3.5)*
Baseline haemoglobin					
<10 gm%	379 (30)	39 (10)	14.5 (10.6–19.8)	1.5 (1–2.2)	1.6 (1.1–2.3)*
⩾10 gm%	760 (60)	62 (8)	10.5 (8.2–13.5)	reference	reference
Not recorded	130 (10)	96 (74)	216.3 (177.1–264.2)	15.2 (11–20.9)**	3.8 (2.3–6.5)**

IHC, Integrated HIV Care; CI, confidence interval; ART, antiretroviral therapy; LTFU, loss to follow-up; MSM, men who sex with men; FSW, female sex worker; MTCT, mother to child transmission; IDU, intravenous drug user; py, person year follow-up.

**P<0.001, *P<0.05.

### ART attrition rates and factors associated with ART attrition

**Fig. 3.**
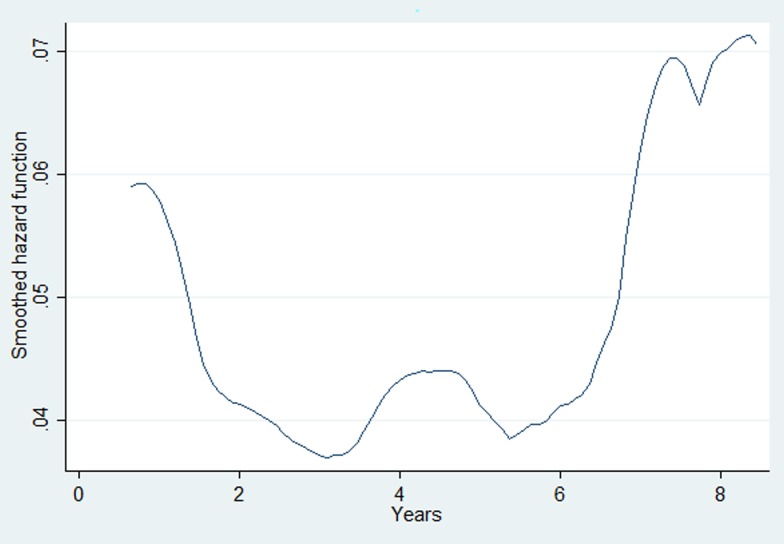
The baseline hazard function for ART attrition derived from the multivariable Cox-proportional hazards model for the adolescent cohort initiated on ART in the Integrated HIV Care Programme, Myanmar (2005–2017). ART, antiretroviral therapy; HIV, human immunodeficiency virus.

The overall attrition rate during the ART period was 6.4 (95% CI 5.6–7.3) per 100 person-years of follow-up. ALHIV who revealed their risk for HIV transmission as intravenous drug users, those had WHO stage 3 and 4, those without documented CD4 cell count test, hepatitis B and C test results at enrolment and those whose haemoglobin was <10 gm/dl had higher hazards of attrition during the ART time periods (Table 3). The baseline hazard function for ART attrition derived from the multivariable Cox-proportional hazards model is given in Figure 3. This indicates that the hazard for attrition in persons on ART is high in the initial 12 months of ART follow-up, then declines, followed by secondary rise after 7–8 years of ART follow-up.

**Table 3.** Socio-demographic and clinical characteristics associated with attrition (during ART period) in adolescents enrolled in IHC Programme, Myanmar from July 2005 to June 2017

Description	Total *n* (%)	Attrition *n* (%)	Attrition rate (100 py) (95% CI)	Unadjusted hazard ratio (95% CI)	Adjusted hazard ratio (95% CI)
Total	1054 (100)	224 (21)	6.4 (5.6–7.3)		
Sex					
Male	538 (51)	114 (21)	6.7 (5.6–8.1)	1.1 (0.8–1.4)	
Female	516 (49)	110 (21)	6.1 (5.1–7.4)	reference	
Entry cohort					
Adult	519 (49)	137 (26)	9 (7.6–10.6)	1.9 (1.5–2.5)**	1.6 (1.0–2.7)
Paediatric	535 (51)	87 (16)	4.4 (3.6–5.4)	reference	reference
Age at enrolment					
10–14 years	572 (54)	101 (18)	4.8 (3.9–5.8)	reference	reference
15–19 years	482 (46)	123 v26)	8.9 (7.4–10.6)	1.7 (1.3–2.2)**	0.9 (0.6–1.4)
Risk factor					
Heterosexual	201 (19)	51 (25)	7.8 (6–10.3)	1.5 (1.1–2)*	1.1 (0.7–1.7)
MSM	13 (1)	3 (23)	9.7 (3.1–30.1)	1.6 (0.5–5.1)	1.7 (0.4–6.4)
FSW	4 (0)	2 (50)	31.7 (7.9–126.6)	4.7 (1.2–19)	2.8 (0.7–11.3)
IDU	46 (4)	11 (24)	12.8 (7.1–23.1)	1.9 (1–3.6)*	2.5 (1.4–4.6)*
Blood Transfusion	38 (4)	6 (16)	4.4 (2–9.8)	0.8 (0.4–1.9)	0.7 (0.3–1.5)
MTCT	639 (61)	118 (18)	5.2 (4.3–6.2)	reference	reference
Unknown	113 (11)	33 (29)	10.3 (7.3–14.5)	1.9 (1.3–2.8)**	1.2 (0.7–1.9)
Baseline WHO stage					
Stage 1	249 (24)	41 (16)	5.8 (4.3–7.9)	reference	reference
Stage 2	229 (22)	24 (10)	3 (2–4.5)	0.6 (0.3–0.9)*	0.6 (0.4–0.9)*
Stage 3	456 (43)	115 (25)	7.2 (6–8.6)	1.4 (0.9–2.0)	1.3 (1.1–1.7)*
Stage 4	116 (11)	40 (34)	10.5 (7.7–14.3)	2.0 (1.2–3.1)*	1.7 (1.2 –2.3)*
Not recorded	4 (0)	4 (100)	104.2 (39.1–277.6)	12.1 (4.3–33.8)**	2.1 (0.5–8.2)
Baseline CD4 (/mm3)					
<200	456 (43)	119 (26)	7.3 (6.1–8.7)	1.5 (1.1–2.2)*	1.4 (0.9–2.1)
200–349	254 (24)	42 (17)	5.0 (3.7–6.8)	reference	reference
350–499	140 (13)	21 (15)	4.6 (3.0–7.0)	0.9 (0.5–1.5)	0.9 (0.7–1.5)
⩾500	184 (17)	26 (14)	4.7 (3.2–6.9)	0.9 (0.6–1.5)	0.9 (0.5–1.6)
Not recorded	20 (2)	16 (80)	106.9 (65.5–174.5)	13.9 (7.8–25)**	8.0 (3.5–18.3)**
Hepatitis B antigen					
Positive	59 (6)	14 (24)	8.1 (4.8–13.7)	1.5 (0.9–2.6)	1.3 (0.7–2.3)
Negative	870 (83)	157 (18)	5.1 (4.4–6)	reference	reference
Not recorded	125 (12)	53 (42)	21.7 (16.6–28.4)	3.6 (2.6–5)*	2.8 (1.6–4.9)**
Hepatitis C antibody					
Positive	45 (4)	6 (13)	5.9 (2.7–13.2)	1 (0.4–2.2)	0.6 (0.2–1.7)
Negative	884 (84)	165 (19)	5.2 (4.5–6.1)	reference	reference
Not recorded	125 (12)	53 (42)	21.7 (16.6–28.4)	3.5 (2.6–4.8)**	2.8 (1.6–4.9)**
Baseline haemoglobin					
<10 gm%	340 (32)	89 (26)	7.9 (6.4–9.7)	1.5 (1.2–2)*	1.4 (1.0–1.9)*
⩾10 gm%	684 (65)	121 (18)	5.2 (4.4–6.2)	reference	reference
Not recorded	30 (3)	14 (47)	28.9 (17.0–48.7)	4.5 (2.6–7.8)**	0.8 (0.3–1.9)
Initiated ART regimen					
AZT-based regimen	270 (26)	54 (20)	5.1 (3.9–6.6)	reference	reference
ABC-based regimen	133 (13)	22 (17)	7.7 (5–11.6)	1.2 (0.7–2.0)	1.4 (0.7–2.8)
TDF-based regimen	361 (34)	76 (21)	9.7 (7.8–12.2)	1.6 (1.1–2.2)*	1.4 (0.9–2.0)
D4T-based regimen	290 (28)	72 (25)	5.3 (4.2–6.6)	1.1 (0.8–1.6)	0.9 (0.5–1.4)

IHC, Integrated HIV Care; CI, confidence interval; AZT, zidovudine; ABC, abacavir; TDF, tenofovir; D4T, stavudine; ART, antiretroviral therapy; LTFU, loss to follow-up; MSM, men who sex with men; FSW, female sex worker; MTCT, mother to child transmission; IDU, intravenous drug user; py, person year follow-up.

**P<0.001, *P<0.05.

## Discussion

This is the first study from Myanmar describing the retention of adolescents on HIV care over a 12-year period. The major findings of this study are: (a) about 16% of ALHIV enrolled as adolescents in the IHC Programme were either LTFU or dead before ART initiation (the pre-ART attrition rate was 21.8 per 100 person-years of follow-up); (b) 21% of the ALHIV who were initiated on ART were either dead or LTFU (ART attrition rates were 6.4 per hundred person-year follow-up); (c) the hazards for pre-ART and ART attrition shows a bimodal curve with higher levels in the initial 1–2 years and after 5–6 years for both pre-ART and ART time periods; (d) a few patient factors (WHO stage, IDU, haemoglobin) were independently associated with pre-ART and ART attrition.

The major strengths of this study were: (a) inclusion of a large number of ALHIV (~15% of ALHIV of Myanmar) over a long follow-up period of 12 years. This has allowed us to measure cumulative attrition rates for various time intervals and also study the baseline hazard function experienced by this cohort both for the short- and long-term; (b) all those who were adolescents at the time of enrolment into IHC Programme were included with no exclusion criteria, data were extracted from the routine programmatic records and therefore the study findings reflect ground-level realities.

The major limitations of this study are: (a) we used baseline/enrolment values for demographic and clinical characteristics. Many of these characteristics are time varying. Therefore, our study reflects the predictors for attrition ‘at enrolment’ values for these characteristics; (b) some of the independent variables (baseline CD4, haemoglobin, hepatitis B and C status) had missing data and those with missing data were associated with pre-ART and ART attrition indicating linkages between missing data and attrition and this would be either due to early attrition before doing these investigations or missed to document results. Hence excluding those cases would have led to selection bias. Instead, we have included those cases by creating a separate category for missing values and estimated the associations. This may have reduced the selection bias, but there may still be biases in our association estimates due to the missing values. At this stage we are unable to speculate whether the association estimates are underestimates or overestimates; (c) as we used the routine programme data, some of the important factors that may have an effect on the outcome such as socio-economic status of the ALHIV households, care giver information, disclosure status, educational status, distance from the ART facilities were not recorded and therefore we are unable to account for these factors in our study. Despite these limitations, we feel the study findings have the following implications on policy and practices.

First, we do not have any similar studies from Asian countries to compare our attrition rates. The overall attrition rate in pre-ART and ART periods in our setting were relatively lower when compared to studies done on adolescents in African countries [3, 19]. The African studies also show higher attrition rates among older adolescents (age ⩾15 years) when compared to younger adolescents (age <15 years) which we did not find in our setting [3, 19]. The lower attrition rates in our setting indicate relatively better organisation and/or delivery of services under the IHC Programme. On the other hand, the pre-ART and ART attrition rates were higher than the attrition rates among paediatric patients in the IHC Programme [18]. This calls for focused attention towards providing additional care to ALHIV under IHC. With the adoption of ‘test and treat’ approach in Myanmar in 2017–18 [17], pre-ART attrition should virtually disappear from our setting and early ART initiation should improve the survival of ALHIV in the future.

Second, baseline hazards for pre-ART and ART attrition were high during the initial 1–2 years and after 5–6 years. High hazards during the initial time periods are due to late stage enrolment (~50% of the adolescents in our study were in WHO stage III, IV and CD4 count of <350 cells/mm^3^ and about 30% had baseline haemoglobin <10 gm/dl). Current comprehensive initial counselling and peer support should be assessed and strengthened for reducing LTFU during initial follow-up visits. Therefore, efforts should be made to detect these cases early and enrol them on ART care. The secondary peak in hazards of attrition found 5–6 years after enrolment indicates waning immunity due to lack of ART or to reduced effectiveness of ART due to undetected virological failure or inadequate provision of psychosocial counselling services tailored to the needs of the adolescent age group. Previous studies show that ALHIV encounter stigma and discrimination, ridicule from their friends/peers that hinders regular follow-up on ART [20]. Assessing and addressing context-specific reasons for late enrolment, presence or absence of virological failure and other psychosocial aspects that hinder adolescents from continuing on ART in our setting is an area for further research.

Third, we found that around 20% of the ALHIV did not have documented investigation/test results for CD4 cell count, hepatitis B, hepatitis C and haemoglobin at enrolment indicating missed opportunities for identifying cases with severe immunodeficiency and/or co-morbidities early. If these baseline investigators were done on time (at enrolment) and if appropriate care (e.g. early ART initiation) was given to those with severe immune deficiency, the survival of adolescents may have been higher.

Lastly, injection drug users (IDUs) had 3.3 times (95% CI 1.3–8.6) and 2.5 times (95% CI 1.4–4.6) higher hazard of pre-ART and ART attritions when compared to other risk groups. This indicates issues related to linkage to IDU substitution therapy centres and dose adjustment for both ART and methadone/naloxone. A study showed that compared to sexually transmitted HIV, IDUs had a higher risk of late presentation, delayed ART initiation and higher mortality [21]. Therefore, in addition to linkages to methadone maintenance programme, further efforts must be made to provide enhanced psychosocial support to address attrition-related issues for these specific ALHIV. Several studies have shown an independent association between attrition and WHO staging 3 or 4 and anaemia [22–24]. We found a similar association in our cohort with those having bad clinical conditions at enrolment (WHO stage 3 or 4 and haemoglobin <10 gm/dl) having a relatively higher hazard of attrition. Our findings support the importance of prioritisation and appropriate management of HIV-infected patients with anaemia and higher WHO clinical stages.

In conclusion, the pre-ART and ART attrition rates in ALHIV were lower than those reported elsewhere in Africa but higher than the children receiving ART care under IHC. This warrant designing and delivering additional care tailored to the needs of ALHIV in our setting such as differentiated adolescents/youth-friendly clinics, transition clinics, youth club activities, integrated or one-stop ART, harm reduction and methadone maintenance services for adolescent IDUs.

## References

[1] UNAIDS (2018) Unaids data 2018. Published online: 2018.

[2] UNAIDS (2017) UNAIDS estimates, 2017. Published online: 2017.

[3] Auld AF (2014) Antiretroviral therapy enrollment characteristics and outcomes among HIV-infected adolescents and young adults compared with older adults – seven African countries, 2004–2013. MMWR. Morbidity and Mortality Weekly Report United States 63, 1097–1103. PMC5779521 25426651

[4] Dahourou DL (2017) Transition from paediatric to adult care of adolescents living with HIV in sub-Saharan Africa: challenges, youth-friendly models, and outcomes. Journal of the International AIDS Society Switzerland 20, 21528. 10.7448/IAS.20.4.21528 PMC5577723 28530039

[5] Pettitt ED (2013) Improving health services for adolescents living with HIV in sub-Saharan Africa: a multi-country assessment. African Journal of Reproductive Health Nigeria 17, 17–31. 24689314

[6] Fatti G (2018) The effectiveness and cost-effectiveness of community-based support for adolescents receiving antiretroviral treatment: an operational research study in South Africa. Journal of the International AIDS Society 21, e25041. 10.1002/jia2.25041 PMC5978711 29485714

[7] Slogrove AL (2018) The epidemiology of adolescents living with perinatally acquired HIV: a cross-region global cohort analysis. PLoS Medicine United States 15, e1002514. 10.1371/journal.pmed.1002514 PMC5832192 29494593

[8] Boerma RS (2017) Multicentre analysis of second-line antiretroviral treatment in HIV-infected children: adolescents at high risk of failure. Journal of the International AIDS Society Switzerland 20, 21930. 10.7448/IAS.20.1.21930 PMC5640308 28953325

[9] Kyaw NTT (2017) High rate of virological failure and low rate of switching to second-line treatment among adolescents and adults living with HIV on first-line ART in Myanmar, 2005–2015. PLoS ONE 12, e0171780. 28182786 10.1371/journal.pone.0171780 PMC5300167

[10] Ministry of Labour I and P. *Population of Myanmar* (https://www.dop.gov.mm/en).

[11] Ministry of Immigration (2014) *Myanmar census report*.

[12] World Health Organization (2006) Antiretroviral Therapy for HIV Infection in Adults and Adolescents: Recommendation for a Public Health Approach. Geneva, Switzerland: WHO publications Published online: 2006. 23741771

[13] World Health Organization (2006) Antiretroviral therapy of HIV infection in infants and children: towards universal access. 1–152. 23741772

[14] National AIDS Programme Myanmar (2011) Guidelines for the clinical management of HIV infection in children. Ministry of Health, 110, 3rd Edn.

[15] National AIDS Programme Myanmar (2011) Guidelines for the Clinical Management of HIV Infection in Adults and Adolescents in Myanmar, 3rd Edn 1–79. Myanmar: Ministry of Health.

[16] National AIDS Programme Myanmar (2014) The Clinical Management of HIV Infection in Myanmar Guideline, 4th Edn Myanmar: Ministry of Health Published online: 2014.

[17] National AIDS Programme Myanmar (2017) Guidelines for the clinical management of HIV infection in Myanmar. Published online: 2017.

[18] Minn AC (2018) Attrition among HIV positive children enrolled under integrated HIV care programme in Myanmar: 12 years cohort analysis. Global Health Action Taylor & Francis, 11, 1–11. Published online: 2018. doi: 10.1080/16549716.2018.1510593. 30191749 PMC6136349

[19] Kranzer K (2017) Loss to follow-up among children and adolescents growing up with HIV infection: age really matters: age. Journal of the International AIDS Society 20, 1–7. 10.7448/IAS.20.1.21737 PMC5577636 28715158

[20] Nabukeera-Barungi N (2015) Adherence to antiretroviral therapy and retention in care for adolescents living with HIV from 10 districts in Uganda. BMC Infectious Diseases BMC Infectious Diseases 15, 1–10. 26573923 10.1186/s12879-015-1265-5 PMC4647509

[21] Suarez-Garcia I (2016) Clinical outcomes of patients infected with HIV through use of injected drugs compared to patients infected through sexual transmission: late presentation, delayed anti-retroviral treatment and higher mortality. Addiction (Abingdon, England) England 111, 1235–1245. 10.1111/add.13348 26890155

[22] Thida A (2014) Retention and risk factors for attrition in a large public health ART program in Myanmar: a retrospective cohort analysis. PLoS ONE 9, 1–12. Published online: 2014. doi: 10.1371/journal.pone.0108615. PMC4182661 25268903

[23] Cuong DD (2012) Survival and causes of death among HIV-infected patients starting antiretroviral therapy in north-eastern Vietnam. Scandinavian Journal of Infectious Diseases England 44, 201–208. 10.3109/00365548.2011.631937 22122590

[24] Argemi X (2012) Impact of malnutrition and social determinants on survival of HIV-infected adults starting antiretroviral therapy in resource-limited settings. AIDS (London, England) 26, 1161–1166. 10.1097/QAD.0b013e328353f363 22472856

